# Accuracy assessment of digital impressions with varied scanning paths in partially edentulous ridges with mobile abutment teeth: An in vitro comparative study

**DOI:** 10.1371/journal.pone.0327380

**Published:** 2025-07-31

**Authors:** Samar S. Alaghbari, Esraa A. M. Saeed, Lu Zhen, Mohammed M. Al Moaleem, Hongjie Chen, Bayan M. Gashan, Shaojie Dong, Lin Niu, Bo Hu

**Affiliations:** 1 Key laboratory of Shaanxi Province for Craniofacial Precision Medicine Research, College of Stomatology, Xi’an Jiaotong University, Xi’an, Shaanxi Province, China; 2 Department of Prosthodontics, College of Stomatology, Xi’an Jiaotong University, Xi’an, Shaanxi Province, China; 3 School and Hospital of Stomatology, Wenzhou Medical University, Wenzhou, Zhejiang, China; 4 Department of Prosthetic Dental Science, College of Dentistry, Jazan University, Jazan, Saudi Arabia; 5 Xi’an Kangtaijian Dental Technology Co., Ltd, Xi’an, Shaanxi Province, China; Kuwait University, Faculty of Dentistry, KUWAIT

## Abstract

**Background:**

Periodontal prosthesis or removable partial dentures are essential treatments for partially edentulous dentition with periodontal issues. This study aimed to assess the accuracy of digital impressions obtained through an intra-oral scanner, employing different scanning paths versus conventional impressions in partially edentulous ridges with mobile abutment teeth.

**Methods:**

Eight lower Kennedy class I and class III models were employed as test models. The abutment teeth in these models were subjected to various mobility grades, according to the Miller classification. Reference data was generated by scanning the test models using an extra-oral laboratory scanner. An intra-oral scanner (TRIOS 4; V21; 3Shape A/S) was used to obtain ten digital impressions following two different scanning paths (Scan path A and Scan path B). For conventional impressions, two impression materials (Monophase polyether and Polyvinyl siloxane) were used to create ten impressions with a one-step technique. Working stone casts were produced and converted to digital data. Accuracy was assessed by analyzing the deviation between test data (digital and conventional data) and the reference data using 3D software (Geomagic Control X). The data was analysis using sequential tests, including two-way and one-way ANOVA, and paired *t*-tests (*p* < 0.05).

**Results:**

Digital impressions obtained through an intra-oral scanner exhibited significantly higher accuracy. Within the digital impression category, those recommended by the manufacturer obtained using scan path A showed lower deviations than those acquired through scan path B. Considering the degree of tooth mobility, models with GII and GIII mobile RPD abutment teeth displayed significantly higher deviations (*p* < 0.001) than those with G0, GI across all impression techniques. The accuracy of conventional impressions with GII and GIII mobility was clinically unacceptable (deviation >200µm).

**Conclusion:**

For partially edentulous cases with mobile abutment teeth, digital impressions exhibited superior accuracy for G0, GI. Following the manufacturer-recommended scanning protocol in scan path A can improve the accuracy of impressions. Furthermore, if there is persistent mobility, particularly in GII and GIII, the use of final conventional impressions is forbidden.

## Introduction

Periodontitis is a prevalent global chronic condition, impacting over 65% of the populace. Beyond its consequences of functional issues, aesthetic concerns, and patient discomfort, advanced periodontal diseases and aggressive forms of periodontitis often culminate in tooth mobility and subsequent loss due to the degradation of the tooth’s periodontal tissues [1].

Many individuals with periodontitis require prosthetic replacement due to tooth loss. As a result, prosthodontists may encounter a more significant number of partially edentulous patients with varying degrees of tooth mobility.

Several studies show that removable partial dentures are strongly recommended for individuals with teeth mobility where the movement is ≥ 1 mm [2–4]. However, when dealing with cases involving mobile teeth, the process of impression-taking, the pursuit of accurate dental casts, and achieving the optimal fit of dental prosthesis become notably more complex [5].

Obtaining an accurate conventional impression for prosthetic purposes is challenging in patients with periodontal issues due to tooth mobility, irregularities, undercuts, and interdental space [6]. The elasticity of the material is often insufficient for the necessary removal forces, leading to tearing and distortion when removing the impression [6]. Separating the impressions can also result in increased tooth mobility, inaccurate representation of tooth position and surrounding anatomical structures, patient discomfort, and potential risk of extracting extremely mobile teeth.

Recently, digital dentistry has seen remarkable advancements, introducing digital intra-oral scanners as a viable alternative to conventional impression methods.

In prosthodontics, numerous studies have examined the accuracy (both trueness and precision) of intra-oral scanners (IOSs) for capturing full-arch impressions in partially and completely edentulous ridges [7–12] and have shown equal or even superior accuracies for the IOSs compared the conventional impressions. However, there is a notable gap in the literature regarding assessing the accuracy of the digital full-arch impressions for partially edentulous ridges featuring mobile teeth.

Therefore, this in vitro study aimed to fill this gap by evaluating the accuracy of digital and conventional impressions in Kennedy class I and class III modifications one with varying degrees of tooth mobility. To offer dental professionals’ evidence- based insights to assist them in choosing the most appropriate impression techniques for cases involving mobile teeth. The null hypothesis of this study was that there is no significant difference in the accuracy between the different degrees of teeth mobility according to the impressions technique and that there is no significant difference in the accuracy between conventional and digital impressions of partially edentulous mandibles with mobile abutment teeth.

## Materials and methods

### Prepare of models

**Fig 1:**
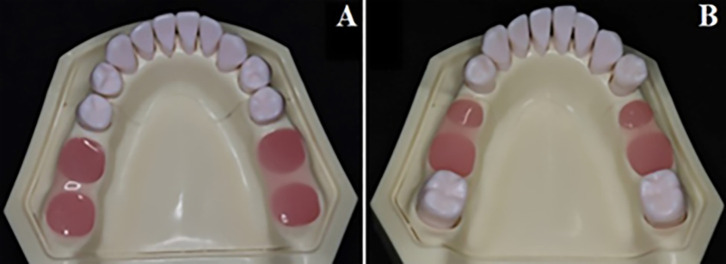
(A) Mandibular model of Kennedy class I, (B) Mandibular model of Kennedy class III modification 1.

In this present study, eight artificial mandibular models (D16FE-500A (GSF)-QF, Nissin Dental Products) equipped with pink gingival silicone (500A-F-GSF) and melamine resin teeth (A5A500; Nissin) securely attached with apical fixation screws were used. Two different groups were created according to the Kennedy classification of missing teeth: group A Kennedy class I models created by removing teeth numbers #36, #37, #46, and #47, and group B Kennedy class III modification one created by removing teeth number #35, #36, #45, and #46. Subsequently, the sockets of the removed teeth were filled with a Silicone-based gingival mask (Gingifast Elastic, Zhermack) and 0.5mm covered the surface of dentulous areas to replicate the gingival contour as seen in (Fig 1).

### Simulation of teeth mobility

In this study, three methods were utilized to replicate the mobility of abutment teeth in the models, varying based on the extent of tooth mobility, including socket enlargement, screws loosening, and a combined approach, incorporating both socket enlargement and screw loosening. The selection of these methods was applied with minor modifications based to previous studies reproduced tooth mobility in laboratory [13–17]. In the models representing Kennedy class I, mobility was created in the second premolars on both the right and left sides. As for the models representing Kennedy class III modification one, mobility was created in the first premolars and second molars on both the right and left sides. The degree of tooth mobility was guided according to Miller classification [18], which includes four grades 0, I, II, and III. Each group in the study exhibited tooth mobility based on these grades. While Grade III teeth are often considered for extraction in clinical practice, their inclusion in this study was essential to provide a comprehensive assessment of impression accuracy across the full spectrum of mobility, reflecting real-world clinical challenges

**No Mobility (G0).** The abutment teeth were securely stabilized on the models with a spring washer and apical screw using a screwdriver. Subsequently, the stability of the fixed teeth was assessed later to verify compliance with the criteria for mobility grade.

**Mobility grade I (GI).** The mobility was simulated through the loosening of screws. A screwdriver was used to loosen the screws until a simple movement began to occur; five full turns were sufficient to create simple mobility in the teeth, which was later measured to ensure that it met the mobility grade criteria. Moreover, to prevent any vertical movement of the teeth after achieving the GI mobility. The screw heads, which protruded approximately 0.3 mm above the base of the models, were covered by applying a layer of hard wax.

**Fig 2:**
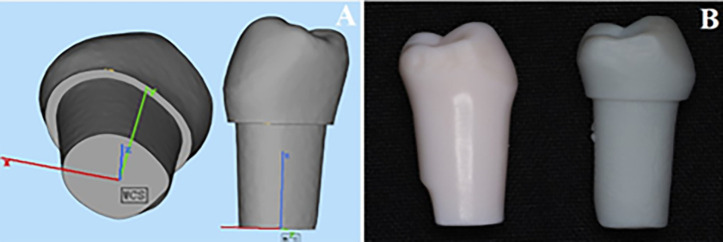
(A) Digital designing of premolar to decrease 1 mm of root surface, (B) 3D printed tooth compared to artificial tooth of model.

**Mobility grade II (GII).** In models of GII, necessitating horizontal movement, either buccolingual or mesiodistal, the socket enlargement method was employed. To establish an accurate space between the socket and the tooth’s root, 3D-printed teeth were substituted for the abutment teeth in the models, designed with a root width 1 mm narrower than the abutment teeth created through the CAD/CAM technique (Fig 2). Subsequently, these 3D-printed teeth were securely affixed in position using a spring washer and an apical screw.

**Mobility grade III (GIII).** In GIII, where horizontal and vertical movements were necessary, a combination approach of socket enlargement and screw loosening, similar to what was described in mobility GI and GII, was employed, and the screw heads were left uncovered by wax to create simple vertical movement.

Furthermore, in cases with GI, a layer of rubber foam with a thickness of 0.3 mm was added to the inner surface of the socket before loosening of screw to duplicate the properties of the periodontal ligament (PDL) of teeth. In GII and GIII cases, a thickness of 1 mm was employed.

### Teeth mobility measurement

The teeth mobility observed in the models corresponding to Miller’s classification was evaluated using the Periotest M device (Periotest, Siemens; AG). The assessment is based on periotest values (PTVs) that range from -8 to +50 [19]. The mobility of each tooth was measured and evaluated three times. However, if the initial mobility by screw loosening did not meet the tooth GI or GIII criteria, the screw loosening was re-adjusted, and a new periotest value was obtained. The results of the PTV measurements were as follows: G0 had a PTV of 3±2, GI had a PTV of 15±1, mobility GII had a PTV of 26±1, and GIII had a PTV of 35±1 with 0.3 mm vertical movement measured manually using digital caliper (LOUISWARE; RO-04; 0.02 mm accuracy).

### Size of sample

The sample size for evaluating the trueness outcome in this study was similar to the one used in pervious study that assessed the trueness of digital and conventional impressions for Kennedy class I and class III cases [20]. This study focused on an effect size of 20µm. To achieve a power of 90% with α = 0.05 and a standard deviation of 8µm, the calculated sample size for an effect size of 19µm was determined to be five impressions for each model. While there is some inconsistency with the previous study, our decision to stick with a sample size of five impressions was influenced by the presence of teeth mobility, as increasing the number of impressions could potentially compromise the accuracy of the impressions.

### Study procedures

**Fig 3:**
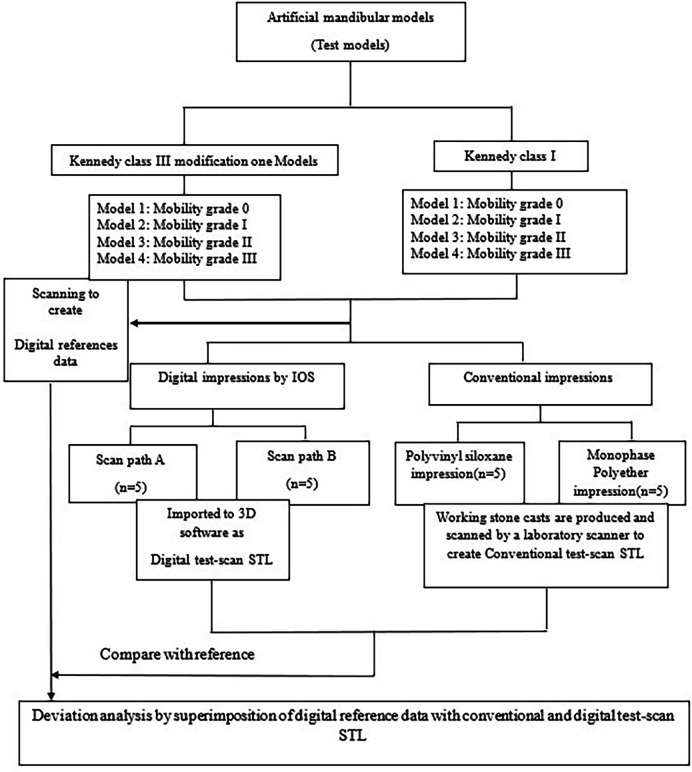
Schematic diagram of study procedures, n = number of impressions per model.

The study groups were divided into two groups based on the impression-making technique. The first was the digital group which was further divided into two subgroups based on the scanning sequence. The second group was the conventional group, which was also divided into two subgroups based on the technique and material of the impression; the schematic diagram of study procedures is illustrated in (Fig 3).

### Digital data

**Fig 4:**
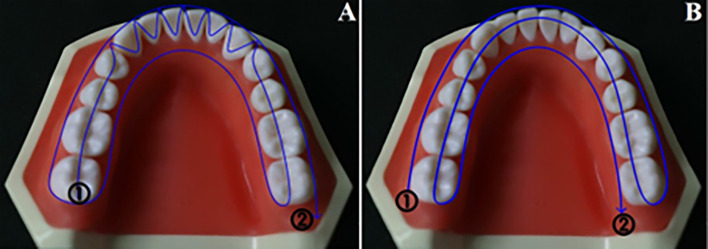
Scanning paths. (A) Scan path A, (B) Scan path B.

For digital group, the class I and class III models were first scanned using 3D laboratory scanner (3Shape model scanner, E4; WI-SB-84) to generate the digital reference data. Subsequently, all eight models were scanned using an intra-oral scanner (TRIOS 4; V21; 3Shape A/S), following two different scanning paths labelled A and B (Fig 4):

Scan path A, as recommended by the manufacturer, involves a continuous scanning process. Scan A starts by scanning the occlusal surface of the lower left posterior area, then moves to the anterior teeth in a labio-lingual pattern. The scan proceeds to the occlusal surface of the lower right posterior area before transitioning from the lingual surface on the right side to the left side. Finally, it concludes by scanning the right buccal surface.

Scan B involves a continuous left-to-right motion, beginning with scanning the buccal surface of the left posterior area, capturing occlusal surfaces, and ending by scanning the lingual surface of the right posterior area. During the scanning process, the models were securely placed on the on the phantom head to simulate the teeth position in clinical setting and to prevent any unintentional movement that could compromise accuracy and reproducibility of impressions.

The scanning procedure followed the guidelines provided by the manufacturer. All scans were conducted under consistent room lighting conditions, utilizing stable and indirect illumination to prevent direct light sources or shadows that might disrupt the scanning process and impact trueness of impressions [21]. After each scan, the models were removed and repositioned to prevent using any past information, and the scanner was switched off between the scans [22]. The digital impressions data was subsequently exported as standard tessellation language (STL) format, saved as digital test-scan STL files (n = 80).

### Conventional data

Two conventional impressions were taken in a single-step technique using Monophase Polyether impression material (3M™ ESPE™ Impregum™ Penta™ Soft Medium Body Polyether Impression Material) and one-step dual-phase polyvinyl siloxane impression material (3M™ ESPE™; Imprint TM 4; Penta TM, Heavy; VPS Impression Material). Also, before applying conventional impressions, the models were placed on a virtual phantom head to approximate the clinical position of teeth.

**Fig 5:**
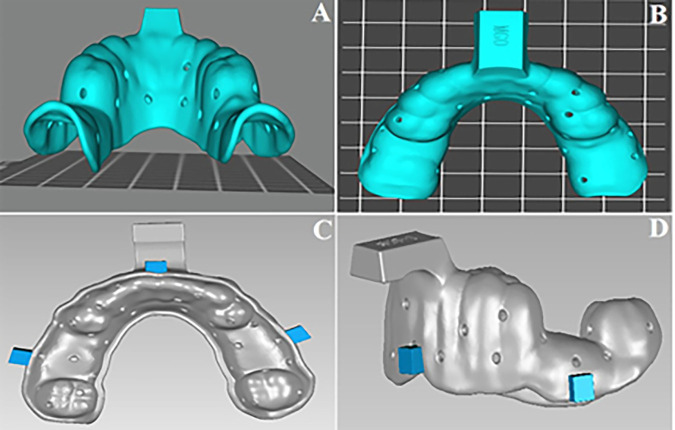
(A) and (B) Digital design of custom tray in posterior view and overhead view, (C) and (D) internal and side view of stop points fabricated in the external surface of the custom tray.

In the single-viscosity monophase technique, to ensure a uniform custom tray for all models and a homogenous distribution of space between the labial and lingual sides of mobile teeth, the custom trays were fabricated digitally using a CAD/3D printer. Three external stop points were added to the custom tray to stabilize the custom tray, ensure precise seating, and control the pressure and force applied during the impression process (Fig 5). The elastomeric polyether impression material was automatically mixed using a Pentamix device (3M ESPE). The impressions were handled according to the manufacturer’s instructions, and the entire process was conducted in a controlled environment with a temperature of 22 ± 1°C.

The one-step putty/light-body technique utilized metallic perforated stock trays (L2; Tecnodent; Casalecchio di Reno). This combined impression was then seated on the model and left undisturbed for four minutes. Following each conventional impression, mobile teeth were carefully repositioned to their original alignment. This was done to ensure that any forces applied during the impression process such as compression or removal of the impression material did not result in cumulative positional changes that could affect subsequent impressions. After repositioning, the mobility of each tooth was re-measured to confirm that the mobility grade remained consistent with the baseline measurement taken before the impression. This step ensured that the tooth’s mobility and position were standardized across all impressions. All the conventional impressions were poured with scannable type IV gypsum (Uni- base300) to create stone casts. The resulting stone casts were then subjected to scanning using a 3D laboratory cast scanner (3Shape model scanner; E4; WI-SB-84), Copenhagen, to generate the conventional test-scan STL data (n=80)*.*

### Deviation analysis and accuracy measurement

To conduct the deviation analysis, the study utilized 3D metrology software (Geomagic Control X; V 2020.1.1; Morrisville). The reference data was aligned with both conventional and digital test-scan data using the software program, starting with the “initial alignment” function and then refining the alignment with the “best-fit alignment” function. This process ensured that the two digital models were positioned with minimal surface distance between them [23]. Subsequently, the 3D compare was activated to analyze the deviations between the models. A deviation was represented by a value produced by automatically calculating the root mean square (RMS) of the amount of deviation at each measurement site using a 3D comparison of the color- difference map. High trueness, signifying a high degree of 3D matching between the superimposed data, is achieved when the RMS value is low [21]. To maintain consistency, the values of maximum and minimum color range were set at +50 and −50 µm, respectively, within +10 and −10 µm tolerance rang. The RMS was estimated in micrometers (µm).

**Fig 6:**
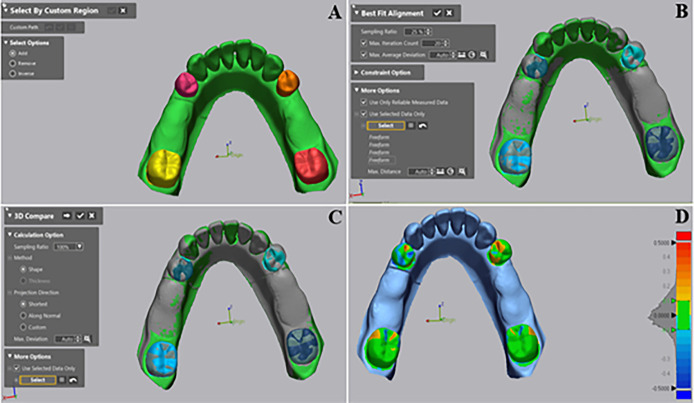
(A) Digital segment of mobile abutment teeth. (B) Data selection of mobile abutment teeth for the best-fit alignment. (C) Data selection of mobile abutment teeth to make a 3D comparison. (D) Color map image of mobile abutment teeth accuracy.

In this study, the RMS values for the two distinct surfaces of the model were computed. These encompassed the surfaces of the mobile abutment teeth and the overall surface. In the overall surface, the STL file of whole models of each test-scan data was superimposed on the STL file of the reference scan data. Color-coded deviation maps were created to visually represent the spatial distribution of discrepancies, allowing for a detailed comparison of deviations between the two distinct datasets. For the deviation analysis of the mobile abutment teeth surface, steps were taken to eliminate errors that may have resulted from the preliminary experiment models’ trimming. The software’s segment tool was initially employed to segment the mobile abutment tooth surface from the entire model. Subsequently, a best-fit alignment was performed by selecting the area corresponding to the mobile abutment teeth using the software’s closest point algorithm, the same range values used for the overall surface were applied to generate color-coded 3D surface deviation maps for calculating RMS values and visually representing the extent of deviation within the mobile abutment teeth area, as illustrated in (Fig 6).

### Statistical analysis

The collected data was analyzed using a statistical software program (IBM SPSS for Windows; V22; SPSS Inc), with a significance level set at 0.05. The Shapiro-Wilks test was employed to verify the normality of the data. Descriptive statistics were computed for variables related to digital and conventional impression techniques within both partially edentulous dentition models. Subsequently, a series of statistical tests were conducted. A two-way ANOVA assessed the interaction between the impression technique and tooth mobility for each evaluated variable. A one-way ANOVA test, followed by the Tukey HSD post hoc test, was utilized to examine differences among various grades of teeth mobility for each technique. A paired *t*-test was also conducted to compare the differences between digital and conventional impression techniques. In this exploratory analysis no multiple testing correction was applied, as this study aimed to identify trends rather than test formal hypotheses.

## Results

### Results of class I models

**Fig 7:**
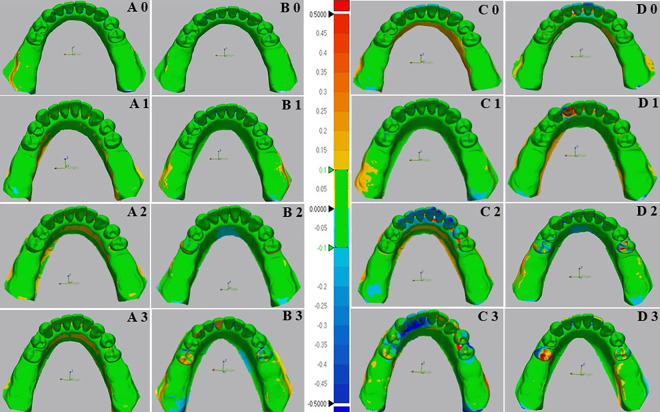
Color images of differences between overall surface of test and reference scanners of digital and conventional impressions for Kennedy class I of the mandibular edentulous ridges. The color difference map is adjusted from −0.5 mm to +0.5 mm. Positive deviations are indicated by yellow to red color, negative deviations by blue color, and nearly no error between two superimposed models by green color. (A) Color images of deviation from digital impressions (Scan path A) and reference models; (B) Color images of deviation from digital impressions (Scan path B) and reference models; (C) Color images of deviation from Conventional impressions (Monophase polyether) and reference models; (D) Color images of deviation from Conventional impressions(Polyvinyl siloxane) and reference models; 0- Indicates mobility grade 0; 1- Indicates mobility grade I; 2- Indicates mobility grade II; and 3- Indicates mobility grade III.

**Fig 8:**
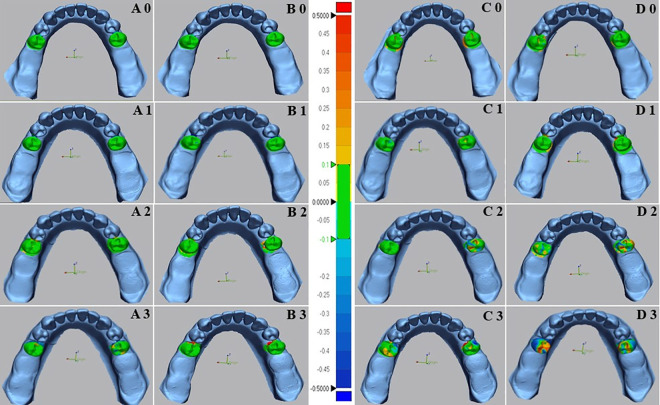
Color images of differences between mobile abutment teeth of test and reference scanners of digital and conventional impressions for Kennedy class I of the mandibular edentulous ridges. The color difference map is adjusted from −0.5 mm to +0.5 mm. Positive deviations are indicated by yellow to red color, negative deviations by blue color, and nearly no error between two superimposed models by green color. (A) Color images of deviation from digital impressions(Scan path A) and reference models; (B) Color images of deviation from digital impressions(Scan path B) and reference models; (C) Color images of deviation from Conventional impressions(Monophase polyether) and reference models; (D) Color images of deviation from Conventional impressions (Polyvinyl siloxane) and reference models; 0- Indicates mobility grade 0; 1- Indicates mobility grade I; 2- Indicates mobility grade II; and 3- Indicates mobility grade III.

The color maps of deviations analysis between conventional and digital tests and reference data of overall surface and mobile RPD abutment teeth surface are shown in (Figs 7 and 8). respectively. The study findings were summarized in (Table 1).

**Table 1:** Summary of the accuracy values (µm) of conventional and digital impressions for each grade of teeth mobility in overall surface and mobile abutment teeth in Kennedy class I models.

Type of impressions		RMS of Overall surface				RMS of Mobile abutment teeth			
	Mobility grade	G0	GI	GII	GIII	G0	GI	GII	GIII
	Mean (SD)	82.8(6.4)	90.4(5.0)	97.6(9.2)	101.8(6.4)	51.8(12.4)	67.2(17.2)	84.6(12.3)	106.2(12.1)
	Maximum	92.0	101.0	111.0	113.0	73.0	94.0	99.0	127.0
	Minimum	76.0	84.0	87.0	97.0	40.0	50.0	67.0	97.0
Digital scan A		All group 0.004:				All group 0.001:			
	P Value	G0 VS. GI= 0.37		GI VS. GII=0.42		G0 VS. GI= 0.32		GI VS. GII=0.22	
		G0 VS. GII=0.02		GI VS. GIII=0.10		G0 VS. GII=0.008		GI VS. GIII=0.002	
		G0 VS. GIII=0.004		GII VS. GIII=0.79		G0 VS. GIII=<0.001		GII VS. GIII=0.10	
	Mean (SD)	85.0(5.3)	97.0(2.6)	98.4(8.2)	114.2(23.6)	57.8(12.6)	90.0(6.6)	93.6(4.1)	107.8(17.3)
	Maximum	92.0	101.0	111.0	156.0	73.0	96.0	99.0	136.0
	Minimum	78.0	94.0	88.0	99.0	45.0	79.0	89.0	94.0
Digital scan B		All group =0.02:				All group <0.001:			
	P Value	G0 VS. GI= 0.47		GI VS. GII=0.99		G0 VS. GI= 0.002		GI VS. GII=0.95	
		G0 VS. GII= 0.38		GI VS. GIII=0.19		G0 VS. GII=0.001		GI VS. GIII=0.10	
		G0 VS. GIII=0.01		GII VS. GIII=0.25		G0 VS. GIII=<0.001		GII VS. GIII=0.24	
Comparison of Digital impressions	Overall Mean	83.9(5.7)	93.7(5.8)	98.0(8.2)	108.0(17.6)	48.1(9.2)	65.2(16.3)	82.9(10.1)	104.1(12.9)
	*P* value	0.66	0.12	0.91	0.35	0.55	0.08	0.22	0.89
	Mean (SD)	87.8(5.8)	173.0 (8.5)	215.0(11.6)	232.2(22.7)	55.0(6.5)	135.6(19.37)	230.4(9.8)	264.0(11.9)
	Maximum	97.0	183.0	231.0	265.0	65.0	162.0	244.0	281.0
	Minimum	82.0	162.0	201.0	207.0	47.0	115.0	217.0	252,0
Monophase		All group <0.001:				All group <0.001:			
	*P* Value	G0 VS. GI= <0.001		G I VS. GII=0.001		G0 VS. GI= <0.001		GI VS. GII =<0.001	
		G0 VS. GII= <0.001		G I VS. GIII=<0.001		G0 VS. GII=<0.001		GI VS. GIII=<0.001	
		G0 VS. GIII=<0.001		G II VS. GIII=0.23		G0 VS. GIII=<0.001		GII VS. GIII=0.004	
	Mean (SD)	106.2(7.8)	174.6(13.9)	237.2(11.6)	257.8(11.0)	70.4(9.2)	153.8(18.8)	252.2(12.9)	361.4(35.3)
	Maximum	116.0	189.0	253.0	275.0	80.0	175.0	272.0	398.0
	Minimum	96.0	159.0	223.0	248.0	56.0	126.0	239.0	312.0
Polyvinylsiloxane		All group <0.001:				All group <0.001:			
	*P* Value	G0 VS. GI= <0.001		GI VS. G II= <0.001		G0 VS. GI=<0.001		GI VS. GII=<0.001	
		G0 VS. GII = <0.001		GI VS. G III= <0.001		G0 VS. GII= <0.001		GI VS. GIII=<0.001	
		G0 VS. GIII= <0.001		GII VS. G III=0.49		G0 VS. GIII= <0.001		GII VS. GIII=<0.001	
Comparison of Conventional impressions	Overall Mean	97.0(11.6)	173.8(10.9)	226.1(16.0)	245.0(21.5)	62.7(11.0)	144.7(20.4)	241.3(15.7)	312.7(57.0)
	*P* value	0.02	0.87	0.01	0.09	0.02	0.32	0.06	0.008
*P* value between Conventional and Digital impressions		0.01	<0.001	<0.001	<0.001	0.09	<0.001	<0.001	<0.001
P value of interaction between impressions techniques and abutment teeth mobility		0.008	<0.001	<0.001	<0.001	0.25	<0.001	<0.001	<0.001

SD: Standard deviation.

**Fig 9:**
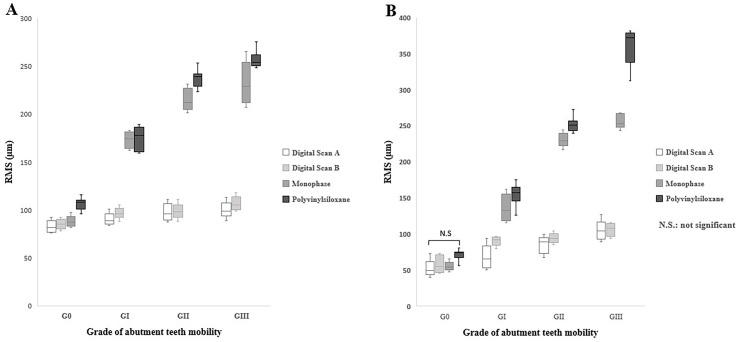
Box plot diagrams of the significant interaction between impressions techniques and abutment teeth mobility in Kennedy class I models. (A) The overall surface, (B) Mobile abutment teeth.

The results of the two-way ANOVA test revealed a significant interaction between the impression technique and the grade of abutment teeth mobility for the overall surface (Fig 9A). The *p* values were (*p* < 0.008) for G0 and (*p* < 0.001) for GI, GII, and GIII. In the case of mobile abutment teeth (Fig 9B), the two-way ANOVA also indicated a significant interaction between the impression technique and the grade of abutment teeth mobility (*p* < 0.001) for GI, GII, and GIII, but not for G0 (*p* = 0.25).

The accuracy of impressions in different grades of teeth mobility showed that digital impressions with scan path A exhibited the lowest deviation values (highest accuracy) for all grades of teeth mobility, followed by scan path B, conventional monophase, and polyvinyl siloxane impressions, respectively, across the overall surface and the mobile abutment teeth.

The one-way ANOVA test indicated highly significant differences between grades of abutment teeth mobility in all impression techniques. The *p* values were (*p* = 0.004) for digital impression with scan path A, (*p* = 0.02) for scan path B, and (*p* < 0.001) for the conventional impressions with monophase and the polyvinylsiloxane material, with the lowest deviation observed in G0, followed by GI, GII, and GIII, which showed the highest deviation across all impressions.

The comparison between digital impression techniques showed no significant difference between impressions of scan path A and scan path B for all grades of teeth mobility. In which the lowest deviation was in G0 (83.9 ± 5.7µm), (48.1 ± 9.2µm) followed by GI (93.7 ± 5.8µm), (65.2 ± 16.3µm), GII (98.0 ± 8.2µm), (82.9 ± 10.1µm), and GIII (108.0 ± 17.6µm), (104.1 ± 12.9µm) in the overall surface and the mobile abutment teeth respectively.

Comparing conventional impression techniques in the overall surface and mobile abutment teeth revealed a significant difference between monophase and polyvinyl siloxane impressions except for GI (*p* = 0.87) and GIII (*p* = 0.09) in the overall surface and GI (*p* = 0.32) and GII (0.06) in the mobile abutment teeth that showed no significant differences present. The lowest deviation among conventional impressions found in G0 (97.0 ± 11.6µm), (62.7 ± 11.0µm), followed by GI (173.8 ± 10.9µm), (144.7 ± 20.4µm), GII (226.1 ± 16.0µm), (241.3 ± 15.7µm), and GIII (245.0 ± 21.5µm), (312.7 ± 57.0µm) across both impression techniques in the overall surface and the mobile abutment teeth respectively.

Across both overall surface and mobile abutment teeth, the comparison between conventional and digital impressions illustrated a highly significant difference (*P* < 0.001) in all grades of teeth mobility except for G0, which showed low significant difference (*P* = 0.01) in the overall surface and no significant difference (*P* = 0.09) in the mobile abutment teeth.

### Results of class III models

**Fig 10:**
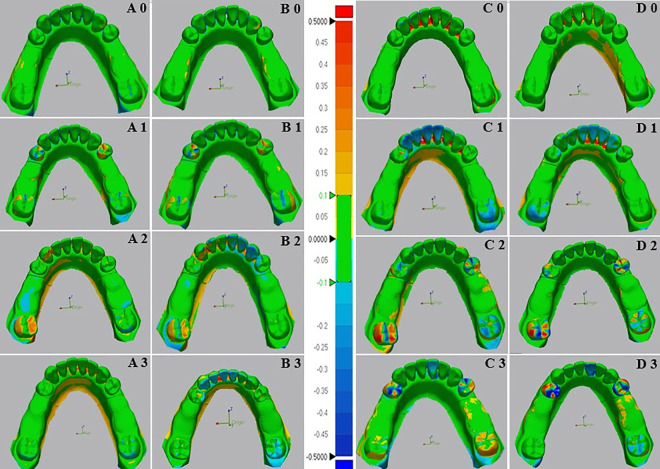
Color images of differences between overall surface of test and reference scanners of digital and conventional impressions for Kennedy class III modification one of the mandibular edentulous ridges. The color difference map is adjusted from −0.5 mm to +0.5 mm. Positive deviations are indicated by yellow to red color, negative deviations by blue color, and nearly no error between two superimposed models by green color. (A) Color images of deviation from digital impressions(Scan path A) and reference models; (B) Color images of deviation from digital impressions(Scan path B) and reference models; (C) Color images of deviation from Conventional impressions(Monophase polyether) and reference models; (D) Color images of deviation from Conventional impressions (Polyvinyl siloxane) and reference models; 0- Indicates mobility grade 0; 1- Indicates mobility grade I; 2- Indicates mobility grade II; and 3- Indicates mobility grade III.

**Fig 11:**
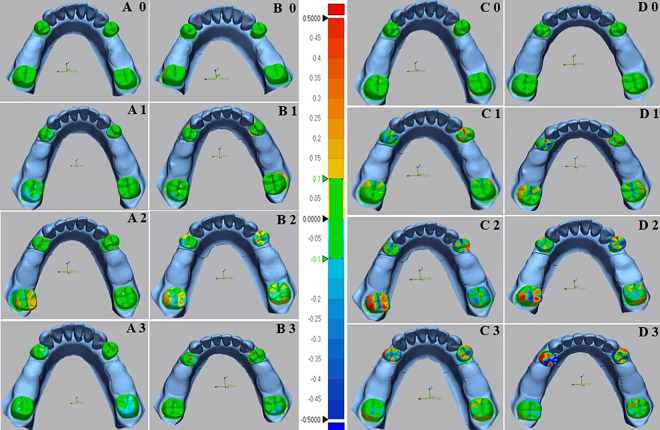
Color images of differences between mobile abutment teeth of test and reference scanners of digital and conventional impressions for Kennedy class III modification one of the mandibular edentulous ridges. The color difference map is adjusted from −0.5 mm to +0.5 mm. Positive deviations are indicated by yellow to red color, negative deviations by blue color, and nearly no error between two superimposed models by green color. (A) Color images of deviation from digital impressions (Scan path A) and reference models; (B) Color images of deviation from digital impressions (Scan path B) and reference models; (C) Color images of deviation from Conventional impressions (Monophase polyether) and reference models; (D) Color images of deviation from Conventional impressions (Polyvinyl siloxane) and reference models; 0- Indicates mobility grade 0; 1- Indicates mobility grade I; 2- Indicates mobility grade II; and 3- Indicates mobility grade III.

The color maps of deviations analysis in class III models between conventional and digital tests and reference data of overall surface and mobile RPD abutment teeth surface are illustrated in (Figs 10 and 11) respectively. The study findings were summarized in (Table 2).

**Table 2:** Summary of the accuracy values (µm) of conventional and digital impressions for each grade of teeth mobility in overall surface and mobile abutment teeth in Kennedy class III models.

Type of impression		RMS of overall surface					RMS of Mobile abutment teeth		
	Mobility grade	G0	GI	GII	GIII	G0	GI	GII	GIII
	Mean (SD)	81.6(3.2)	89.2(2.5)	97.4(5.5)	102.4(7.7)	45.4(5.8)	54.0(8.7)	76.4(8.9)	99.2(8.2)
	Maximum	85.0	93.0	107.0	113.0	51.0	60.0	89.0	107.0
	Minimum	77.0	86.0	93.0	96.0	36.0	39.0	67.0	86.0
Digital scan A		All group <0.001:				All group <0.001:			
	*P* Value	G0 VS. GI= 0.13		GI VS. GII=0.09		G0 VS. GI= 0.57		GI VS. GII=0.01	
		G0 VS. GII=0.001		GI VS. GIII=0.005		G0 VS. GII=0.001		GI VS. GIII= <0.001	
		G0 VS. GIII=<0.001		GII VS. GIII=0.44		G0 VS. GIII=<0.001		GII VS. GIII=0.001	
	Mean (SD)	87.2(3.8)	95.6(5.0)	102.6(7.4)	111.6(11.3)	50.8(11.7)	76.4(14.5)	89.4(6.7)	109.0(15.8)
	Maximum	93.0	102.0	114.0	129.0	67.0	96.0	99.0	132.0
	Minimum	84.0	89.0	96.0	99.0	36.0	57.0	81.0	90.0
Digital scan B		All group <0.001:				All group <0.001:			
	*P* Value	G0 VS. GI= 0.32		GI VS. GII=0.47		G0 VS. GI= 0.008		GI VS. GII= 0.26	
		G0 VS. GII=0.02		GI VS. GIII=0.01		G0 VS. GII= <0.001		GI VS. GIII= 0.01	
		G0 VS. GIII=0.001		GII VS. GIII=0.26		G0 VS. GIII= <0.001		GII VS. GIII= 0.49	
Comparison of Digital impressions	Overall Mean	84.4(4.4)	92.4(5.0)	100.0(6.7)	107.0(10.3)	48.1(9.2)	65.2(16.3)	82.9(10.1)	104.1(12.9)
	*P* value	0.11	0.13	0.33	0.22	0.42	0.005	0.04	0.41
	Mean (SD)	89.6(4.4)	166.8(29.3)	215.4(41.1)	241.8(29.2)	50.0(9.7)	138.0(21.0)	242.4(30.6)	262.6(20.4)
	Maximum	95.0	185.0	265.0	273.0	63.0	155.0	276.0	289.0
	Minimum	84.0	196.0	155.0	201.0	36.0	102.0	207	237.0
Monophase		All group <0.001:				All group <0.001:			
	*P* Value	G0 VS. GI= <0.001		G I VS. GII=0.001		G0 VS. GI= <0.001		GI VS. GII=<0.001	
		G0 VS. GII= <0.001		G I VS. GIII=0.001		G0 VS. GII= <0.001		GI VS. GIII=<0.001	
		G0 VS. GIII= <0.001		G II VS. GIII=0.99		G0 VS. GIII= <0.001		GII VS. GIII=0.54	
	Mean (SD)	98.8(17.1)	177.8(5.8)	247.4(15.8)	263.4(21.1)	57.0(9.7)	173.4(17.2)	251.3(13.4)	329.8(45.6)
	Maximum	123.0	185.0	260.0	287.0	67.0	198.0	271.0	384.0
	Minimum	77.0	169.0	220.0	232.0	47.0	155.0	233.0	368.0
Polyvinylsiloxane		All group <0.001:				All group <0.001:			
	*P* Value	G0 VS. GI= <0.001		GI vs. GIII=<0.001		G0 VS. GI= <0.001		GI VS. GII= <0.001	
		GI VS. GII= <0.001		G0 VS. GIII= <0.001		G0 VS. GII= <0.001		GI VS. GIII= <0.001	
		G0 VS. GII= <0.001		GII VS. GIII= 0.65		G0 VS. GIII= <0.001		GII VS. GIII= 0.001	
Comparison of Conventional impressions	Overall Mean	92.7(12.2)	172.3(20.7)	241.0(20.9)	249(26.7)	53.9(9.7)	155.7(26.0)	248.1(23.4)	298.4(46.0)
	*P* value	0.54	0.47	0.09	0.06	0.17	0.04	0.47	0.02
*P* value between Conventional and Digital impressions		0.06	<0.001	<0.001	<0.001	0.25	<0.001	<0.001	<0.001
P value of interaction between impressions techniques and abutment teeth mobility		0.51	<0.001	<0.001	<0.001	0.79	<0.001	<0.001	<0.001

SD: Standard deviation.

**Fig 12:**
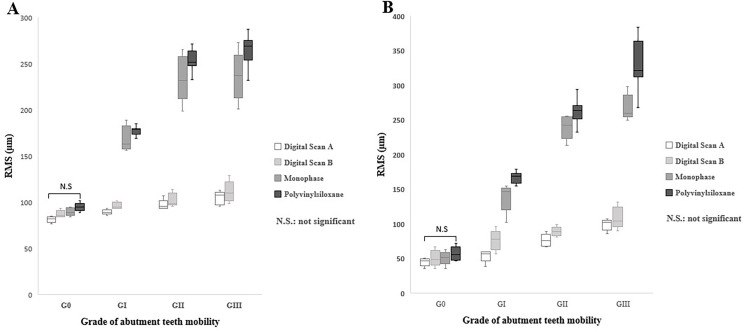
Box plot diagrams of the significant interaction between impressions techniques and abutment teeth mobility in Kennedy class III models. (A) The overall surface, (B) Mobile abutment teeth.

The two-way ANOVA test for the overall surface and mobile abutment teeth revealed a highly significant interaction between the impression technique and all grades of tooth mobility (*p* < 0.001) except for G0, which showed no significant differences (*p* = 0.51) and (*p* = 0.79) in the overall surface and mobile abutment tooth respectively (Fig 12).

The accuracy of impressions in different grades of teeth mobility showed that digital impressions with scan path A exhibited the lowest deviation values (highest accuracy) for all grades of teeth mobility, followed by scan path B, conventional monophase, and polyvinyl siloxane impressions, respectively, across the overall surface and the mobile abutment teeth.

The one-way ANOVA test indicated highly significant differences between grades of abutment teeth mobility in all impression techniques (*p* < 0.001), with the lowest deviation observed in G0, followed by GI, GII, and GIII, which showed the highest deviation across all impressions.

The comparison between digital impression techniques showed no significant difference between scan path A and scan path B for all grades of tooth mobility except for GI (*p* = 0.005) and GII (*p* = 0.04) in the mobile abutment teeth. In which the lowest deviation was in G0 (84.4 ± 4.4µm), (48.1 ± 9.2µm) followed by GI (92.4 ± 5.0µm), (65.2 ± 16.3µm), GII (100.0 ± 6.7µm), (82.9 ± 10.1µm), and GIII (107.0 ± 10.3µm), (104.1 ± 12.9µm) in the overall surface and the mobile abutment teeth respectively.

Comparing conventional impression techniques in the overall surface and mobile abutment teeth revealed no significant difference between monophase and polyvinyl siloxane impressions among all grades of teeth mobility except for GI (*p* =0.04) and GIII (*p* = 0.02) in the mobile abutment teeth. In which the lowest deviation was found in G 0 (92.7 ± 12.2µm), (53.9 ± 9.7µm), followed by GI (172.3 ± 20.7µm), (155.7 ± 26.0µm), GII (241.0 ± 20.9µm), (248.1 ± 23.4µm), and GIII (249 ± 26.7µm), (298.4 ± 46.0µm) across both impression techniques in the overall surface and the mobile abutment teeth respectively.

Furthermore, across both overall surface and mobile abutment teeth, the comparison between conventional and digital impressions illustrated a highly significant difference (*p* < 0.001) in all grades of teeth mobility except for G0, which showed no significant difference (*p* =0.06) in the overall surface and (*p* =0.25) in the mobile abutment teeth.

## Discussions

In the previous studies, various methods were utilized to simulate teeth mobility, broadly categorized into screw loosening, socket enlargement, and a combination of these approaches. Two research studies integrated socket enlargement into their synthetic plastic models, with one employing diamond bur of high- speed to enlarge the artificial socket [16], and the other by replacing the model central incisor tooth with a smaller-sized lateral incisor [17]. However, these studies were considered less clinically accurate due to the imprecise manual techniques employed in socket enlargement [24]. In this study, socket enlargement was refined by reducing the root width of abutment teeth by 1 mm using CAD/CAM techniques, creating accurate space between the socket and the tooth’s root compared to previous studies.

Moreover, the choice of rubber foam over silicone aligns with previous research findings, highlighting its superior ability to closely mimic the natural resetting capabilities of the PDL [15,24]. Additionally, the application of rubber foam helped prevent any irreversible movement of the tooth during impression-making.

The assessment of accuracy in the present study revealed that digital impressions obtained through an intra-oral scanner exhibited significantly lower deviations, indicative of greater accuracy than conventional impressions for mobile abutment teeth in both class I and class III models. Furthermore, within the digital impression category, those obtained using scan path A demonstrated lower significant deviations than those acquired through scan path B. When considering the degree of teeth mobility, models with GIII mobile abutment teeth displayed significantly lower accuracy, than those with teeth mobility G0, GI, and GII in all impression techniques for both missing tooth type models. Consequently, the null hypothesis of this study was rejected.

Comparing the grades of teeth mobility according to the digital impressions demonstrated that digital impressions with scan path A exhibited minimal deviations in G0, GI, GII, and GIII compared to scan path B. This underscores that alterations in the scanning sequence of the IOSs impact impression accuracy, coinciding with previous studies emphasizing that following the manufacturer’s recommended method results in improved impression accuracy [25–28]. Varying positive and negative deviations were also noted in interdental spaces. This resulted from challenges in angulating the scanner handpiece to generate 3D images on the focal plane for interdental spaces, potentially contributing to increased deviations in these areas [6,29].

The evaluation of digital impressions showed that the discrepancy in Kennedy class I is lower compared to Kennedy class III. This finding was consistent with that of a previous study, indicating that the accuracy of digital impressions is influenced by the type of missing tooth and positioning of dentition, where isolating abutment teeth (mesial displacement tooth) with mucosal areas can decrease overall accuracy [28]. Also, the number of mobile teeth that increased deviation in class I was less than in class III.

Generally, errors up to 0.2 mm of RMS value for full-arch impressions have been deemed clinically acceptable, while errors exceeding 0.2 mm have been clinically unacceptable [30–32]. Various studies on partially edentulous jaws indicate that the acceptable deviation is typically less than 200 µm [7,12,20,33]. In the present study, despite the clinical acceptability of digital impressions obtained with scan paths A and scan path B for all grades of teeth mobility (RMS less than 200µm), the deviation values were significantly greater in GIII, where tooth movement was more noticeable than other tooth mobility grades. This discrepancy may be attributed to potential contact between mobile teeth and the scanner head during impression-taking in constrained spaces on the dental operatory chair headrest, resulting in the movement of mobile teeth and errors in the scanned images, ultimately leading to increased deviation.

When assessing the grades of teeth mobility in terms of conventional impression techniques, it was observed that monophase impressions demonstrated minimal deviations in G0, GI, GII, and GIII compared to polyvinyl siloxane. This can be attributed to using a digital custom tray with external stop points, enhancing the impressions’ accuracy [34,35]. Nevertheless, the deviation in all conventional impression techniques for the GII and GIII models exceeded clinically acceptable limits (accuracy exceeding 200µm).

Within the scope of this investigation, conventional impressions also displayed significant variations in tooth mobility grades (*p* < 0.001). Mobile abutment teeth with G0 and GI exhibited lower deviation values, displaying fewer negative and positive deviations in color mapping compared to the GII and GIII. Conversely, GII and G III models revealed considerably higher deviation values in both missing tooth type models. Color mapping illustrated a disproportionate distribution of highly negative and positive deviations on mobile abutment teeth. This inconsistency can be ascribed to the significant movement of mobile teeth during impression-taking, where the deviations were considered unacceptable (errors >0.2mm) [30].

Moreover, color mapping of conventional impressions for overall surface of both partially edentulous dentition models revealed significant negative and positive deviations in the interdental spaces and lingual sides. This occurrence is associated with infiltrating elastomeric impression material into interdental spaces during the impression, creating significant tensile stress and tearing in the interproximal region during removal. These findings were in agreement with the results of a previous study [20], which indicated that during impression removal, the pressure applied to the lingual side of teeth and the alveolar ridge contributed to compression in these areas, resulting in deformity.

While significant differences favoring digital impressions over conventional ones were identified in this research, indicating the superior accuracy and clinical acceptability of digital impressions in the presence of various grades of tooth mobility, it is crucial to exercise caution when implementing digital impression techniques clinically. Though the local trueness and dimensional accuracy of the digital impressions could be impacted as a result of the greater distance from the scanning origin [28], however with the presence of tooth mobility, ensuring an adequate distance between the scanner head and mobile teeth is critical to prevent potential contact that might induce movement of mobile teeth during scanning, leading to errors and loss of impression accuracy.

Limitations of the current study include using a solid plastic typodont with laboratory tooth mobility replication. The investigation did not consider factors associated with the oral environment, such as humidity, resilience, soft tissues, tissues around the mobile teeth, or the effect of PDL. The accuracy of scanners may be affected by these factors.

## Conclusion

Based on the outcomes of this research, the following conclusions can be drawn:

1. Tooth mobility significantly influences both conventional and digital impressions.
2. Digital impressions exhibited superior accuracy than conventional impressions for mobile abutment teeth in both Kennedy class I and class III across various mobility grades.
3. Digital impressions obtained with an intra-oral scanner using scan path A, as recommended by the manufacturer, demonstrated significantly higher accuracy than those obtained with scan path B. This highlights the impact of different scan sequences on impression accuracy for mobile teeth.
4. The assessment of the clinical conventional impression accuracy with GII and GIII mobility was deemed clinically unacceptable.

## Abbreviations

IOSs: Intraoral scanners
CAD/CAM: Computer-aided design/Computer-aided manufacturing
PDL: Periodontal ligament
STL: Standard tessellation language
PTVs: Periotest values
RMS: Root Mean Square
G 0: Grade 0
G I: Grade I
G II: Grade II
G III: Grade III.
